# Noncommunicable diseases and the use of health services: analysis of the National Health Survey in Brazil

**DOI:** 10.1590/S1518-8787.2017051000090

**Published:** 2017-06-01

**Authors:** Deborah Carvalho Malta, Regina Tomie Ivata Bernal, Margareth Guimarães Lima, Silvânia Suely Caribé de Araújo, Marta Maria Alves da Silva, Maria Imaculada de Fátima Freitas, Marilisa Berti de Azevedo Barros

**Affiliations:** I Departamento de Enfermagem Materno Infantil e Saúde Pública. Escola de Enfermagem. Universidade Federal de Minas Gerais. Belo Horizonte, MG, Brasil; II Núcleo de Pesquisas Epidemiológicas em Nutrição e Saúde. Faculdade de Saúde Pública. Universidade de São Paulo. São Paulo, SP, Brasil; III Departamento de Medicina Preventiva e Social. Faculdade de Ciências Médicas. Universidade Estadual de Campinas. Campinas, SP, Brasil; IV Departamento de Vigilância de Doenças e Agravos Não Transmissíveis e Promoção da Saúde. Secretaria de Vigilância em Saúde. Ministério da Saúde. Brasília, DF, Brasil; V Hospital das Clínicas. Universidade Federal de Goiás. Goiânia, GO, Brasil

**Keywords:** Chronic Disease, epidemiology, Health Services, utilization, Health Services Accessibility, Health Services Needs and Demand, Equity in Access, Health Surveys, Doença Crônica, epidemiologia, Serviços de Saúde, utilização, Acesso aos Serviços de Saúde, Necessidades e Demandas de Serviços de Saúde, Equidade no Acesso, Inquéritos Epidemiológicos

## Abstract

**OBJECTIVE:**

To assess whether sex, education level, and health insurance affect the use of health services among the adult Brazilian population with chronic noncommunicable diseases (NCD).

**METHODS:**

Data from a cross-sectional survey were analyzed, the National Health Survey (PNS). Frequency of use of services in the population that referred at least one NCD were compared with the frequency from a population that did not report NCD, according to sex, education level, health insurance, and NCD number (1, 2, 3, 4, or more). The prevalence and prevalence ratios were calculated crude and adjusted for sex, age, region, and 95% confidence intervals.

**RESULTS:**

The presence of a noncommunicable disease was associated with increase in hospitalizations in the last 12 months, in 1.7 times (95%CI 1.53–1.9). Failing to perform usual activities in the last two weeks for health reasons was 3.1 times higher in NCD carriers (95%CI 2.78–3.46); while the prevalence of medical consultation in the last 12 months was 1.26 times higher (95%CI 1.24–1.28). NCD carriers make more use of health services, as well as women, people with higher number of comorbidities, with health insurance, and higher education level.

**CONCLUSIONS:**

NCD carriers make more use of health services, as well as women, people with higher number of comorbidities, with health insurance, and higher education level.

## RESUMO

**OBJETIVO:**

Analisar se sexo, escolaridade e posse de plano de saúde influenciam a utilização de serviços de saúde entre a população adulta brasileira portadora de doenças crônicas não transmissíveis (DCNT).

**MÉTODOS:**

Foram analisados dados de inquérito transversal, a Pesquisa Nacional de Saúde (PNS). Foram comparadas as frequências de uso de serviços na população que referiu pelo menos uma DCNT, com aquelas que não relatam DCNT, segundo sexo, escolaridade, posse de plano de saúde e número de DCNT (1, 2, 3, 4 ou mais). Foram calculadas as prevalências e razões de prevalência (RP) brutas e ajustadas por sexo, idade e região e respectivos intervalos de confiança de 95%.

**RESULTADOS:**

A presença de doença crônica associou-se ao aumento de internação nos últimos 12 meses, em 1,7 vezes (IC95% 1,53–1,9). Deixar de realizar atividades habituais nas duas últimas semanas por motivo de saúde foi 3,1 vezes maior em portadores de DCNT (IC95% 2,78–3,46), e a prevalência de consulta médica nos últimos 12 meses foi 1,26 vezes maior (IC95% 1,24–1,28). Portadores de DCNT utilizam mais os serviços de saúde, assim como as mulheres, pessoas com maior número de DCNT, com planos de saúde e elevada escolaridade.

**CONCLUSÕES:**

Portadores de DCNT têm maior utilização de serviços de saúde, assim como as mulheres, pessoas com maior número de comorbidades, com planos de saúde e elevada escolaridade.

## INTRODUCTION

Noncommunicable diseases (NCD) (cardiovascular diseases, chronic respiratory diseases, diabetes, and cancers) are responsible for about 70% of all deaths worldwide – around 38 million deaths annually. Of these, 16 million deaths occur prematurely (people under 70 years of age) and nearly 28 million in low-and middle-income countries^a,b^.

Evidences indicate an increase in NCD due to the growth of the four main risk factors (smoking, physical inactivity, harmful use of alcohol, and unhealthy diets)^1,a,b,c^. Therefore, intervention in the risk factors would result in the reduction of the number of deaths around the world^a,b,c^.

An epidemic of NCD results in devastating consequences for individuals, families, and communities, and it also overloads health systems^a,b^. Studies show that NCDs are more likely to affect low-income populations, as they are more vulnerable and more exposed to the risk factor, as well as having less access to health services and health promotion and disease prevention practices^2,a^. The World Health Organization (WHO) estimates that people with NCD have their poverty exacerbated by the largest family spending due to the disease, seeking of services, among others^2,b^.

The socioeconomic costs associated with NCD have repercussions on the economies of the countries – estimated at US$7 trillion, for 2011-2025, in countries of low and medium income^a^. Thus, worldwide reduction of NCD is a necessary condition for the development of the 21^st^ century^2,d,e^.

In September 2011, this scenario resulted in the commitment of world leaders at the UN Assembly of defining concrete actions to fight these diseases^f^. In 2013, the World Health Assembly adopted a comprehensive global monitoring program with 25 indicators and nine voluntary goals to 2025, in addition to approving the Global Action Plan for the Prevention and Control of Noncommunicable Diseases 2013-2020^d^. Among the goals set are the reduction of 25% of deaths caused by NCD, reduction of risk factors (smoking, alcohol, salt, physical inactivity), and access to medicines, advice and technologies for treatment of NCDs^d^. Access to assistance by NCD carriers, including medical consultations, primary health care, access to medicines, laboratory tests, clinical practice and counseling, benefits the assistance to NCD carriers and improves quality of life^3^.

WHO highlights that several populations in different countries have hindered access and utilization of health services, which is the main barrier when facing NCD, especially to minimize the suffering of those who are already sick^4,a,b^. Therefore, facing NCD involves governance and public policies and actions aimed at the prevention and reduction of the risk factors, access to health care, organization of surveillance and monitoring, in addition to confronting the social determinants such as reduction of poverty and social inequality, themes that were included in the sustainable development challenges^2,e^.

The use of health services is higher among sick people. Travassos et al.^5^ highlighted that the determinants of use of the services comprehend the health needs or the existence of the disease, as well as its severity and urgency. Other factors of demand for services would be demographic characteristics of users, such as age, sex, region of housing, and socioeconomic status (income, education). Additionally, there are factors related to service providers, such as professional experience, specialty, available resources, geographic and social access, funding, health insurance services, among others^5^.

It is important to monitor the access and use of services among people with NCD, due to the high burden of disease, which has been even more magnified because of population aging. The high use of health services requires planning of services and adequacy of policies and offers. It is essential to know this demand^5-7^.

In 2013, aiming to monitor these themes, Brazil carried out the National Health Survey (PNS), a household and population-based survey that includes topics such as NCD and access and use of services^g^. The questionnaire of the 2013 PNS covered a broader set of NCD, beyond those advocated by WHO^b^– such as back problems, arthritis, rheumatism, and chronic kidney diseases –, as they are high magnitude causes of morbidity, in addition to being responsible for loss of quality of life of the population^g^. 2013 PNS identified that 45% of the Brazilian adult population reported having at least one NCD, the most frequent being: high blood pressure, pain in the spine or back, diabetes, arthritis or rheumatism, depression, and bronchitis or asthma^8,g^.

This study aims to assess whether sex, education level, and health insurance affect health services use among the adult Brazilian population with NCD.

## METHODS

Data from the cross-sectional study PNS, developed by the Brazilian Institute of Geography and Statistics (IBGE) in partnership with the Ministry of Health, were analyzed. This is the most complete research on health and its determinants ever carried out in the Country^9,10^. Counting with its own design, the survey collects information on several aspects of health.

PNS sampling plan was developed by conglomerates in three stages of selection. In the first stage, primary care units (UPA) were selected by simple random sampling, consisting of census tracts or sets of census tracts (when the tracts counted with few households). In the second stage, a fixed number of households were selected by simple random sampling for each UPA, ranging from 10 to 14. In each household sampled, one resident with 18 years or more was selected, also by simple random sampling, to take part in the third stage of selection^9,10^.

Sample was calculated at approximately 80,000 households. Information were collected on 62,986 households. The calculation took into account average values, variances, and the effects of the sampling plan, assuming a nonresponse rate of 20%^9,10,g^.

The information on access to and use of health services were obtained by the household informant (proxy informant), who answered on use of services in the name of all residents. Valid information was collected for 205 thousand residents^g^.

The weights of the households and all its residents were calculated by the product of the weight of the UPA in question and the inverse of the probability of selection of the household within the UPA. The weights were adjusted to correct nonresponses and to calibrate the estimates according to population totals known from other sources. The selection of the resident who answered to the individual interview was done by simple random sampling. Thus, the weight of the selected resident was calculated by the product of the weight of the household by the number of eligible residents (equivalent to the inverse of the probability of selection). More details can be consulted in another study^g^.

Data collection was carried out with the use of handheld computers (personal digital assistant), programmed to critique the received values. The PNS questionnaire is divided in three parts: household information; information of all residents, answered by one resident (proxy); information about the selected resident, answered only by himself/herself (being an adult aged 18 years or more)^9,10,g^.

In the section concerning the selected resident, 60,202 interviews were carried out with the adult selected at the household. This study analyzes information regarding NCDs, one or more diseases referred among the following: hypertension; diabetes; heart disease; stroke; asthma; arthritis or rheumatism; WMSD (work-related musculoskeletal disorder); cancer; chronic renal failure; chronic back problem; depression, or other mental illness; and lung disease (emphysema, chronic bronchitis, or COPD). The question referred to previous medical diagnosis for most NCDs, except in the case of chronic back pain, which was self-reported, and in the case of depression and mental health, which considered previous diagnosis of a physician or mental health professional (such as a psychiatrist or psychologist).

In the case of the proxy informant, in the household questionnaire, questions were made and the respondent answered for all residents. The questions concerned: a) In the last two weeks, did you seek any place, service or health professional for health-related care? (J14); b) In the last two weeks, did you fail to perform any of your usual activities due to your health? (J2); c) When did you last see a doctor? (J11 = 1 = in the last 12 months); d) In the last 12 months, did you stay in the hospital for 24 hours or more? (J37).

Frequency of use of services in the population that referred at least one NCD were compared with the frequency from a population that did not report NCD, according to sex, education level, health insurance, and NCD number or comorbidities (1, 2, 3, 4, or more). The prevalence and prevalence ratios (PR) were calculated crude and adjusted for sex, age, region, and 95% confidence intervals.

As the data was collected using a complex sampling design, the statistical analysis was carried out with an application that takes into consideration the effect of sampling plan and the unequal probabilities of selection. The data were analyzed in the Stata 11.0 software, using the survey mode, which takes into consideration the effects of complex sampling^h^.

The PNS was approved by the National Committee of Ethics in Research (Process: 328,159, June 26, 2013). All individuals were consulted, the survey was clarified to them, and they agreed to participate.

## RESULTS

The study showed that reporting the presence of at least one chronic disease was shown to be associated, to the Brazilian adult population in 2013, with the increased use of health services in the last two weeks, 25.6% in adults with and 10.8% in adults without NCD (adjusted PR = 2.0; 95%CI 1.88–2.18). The presence of chronic disease was associated with: increased hospitalization, 1.7 times over the last 12 months (95%CI 1.53–1.9); failing to perform activities in the last two weeks for health reasons, 3.1 times higher than those who did not report NCD (95%CI 2.78–3.46); and prevalence of medical consultation in the last 12 months, 1.26 times greater (95%CI 1.24–1.28) (Table 1). The same pattern repeats itself with higher PR, when comparing the same indicators, among the population with and without NCD, for both women and men. In other words, the population with NCD uses more health service and fails more frequently to perform activities. Women with and without NCD, compared with men with and without NCD, have higher PR for the use of health services, hospitalization, medical consultation, and failing to perform usual activities for health reasons.

**Table 1:** Use of health services and failing to perform activities for health reasons in the population with and without NCD, according to sex. National Health Survey, 2013.

Variable	Population with NCD		Population without NCD		Crude PR^a^	Adjusted PR^b^	95%CI
Total	%		%				
Use of health services in the last two weeks	25.64		10.87		2.36	2.02	1.88–2.18
Hospitalization in the last 12 months	9.61		4.91		1.96	1.7	1.53–1.9
Failing to perform activities in the last two weeks for health reasons	12.92		4.06		3.18	3.1	2.78–3.46
Medical consultation in the last 12 months	84.96		65.36		1.3	1.26	1.24–1.28
Males	%	Adjusted PR (95%CI)^c^	%	Adjusted PR (95%CI)^c^			
Use of health services in the last two weeks	20.89	1	8.31	1	2.52	2.1	1.86–2.38
Hospitalization in the last 12 months	8.69	1	3.05	1	2.85	2.14	1.78–2.58
Failing to perform activities in the last two weeks for health reasons	10.73	1	3.63	1	2.95	2.73	2.28–3.27
Medical consultation in the last 12 months	78.69	1	56.96	1	1.38	1.31	1.26–1.35
Females							
Use of health services in the last two weeks	28.93	1.38 (1.27–1.51)	13.67	1.65 (1.48–1.84)	2.12	1.96	1.8–2.14
Hospitalization in the last 12 months	10.24	1.18 (1.04–1.34)	6.94	2.28 (1.90–2.74)	1.48	1.5	1.31–1.72
Failing to perform activities in the last two weeks for health reasons	14.45	1.34 (1.20–1.50)	4.53	1.25 (1.04–1.50)	3.19	2.92	2.53–3.38
Medical consultation in the last 12 months	89.31	1.13 (1.11–1.16)	74.51	1.31 (1.27–1.35)	1.2	1.18	1.16–1.2

^a^ Crude PR, comparing use of health services according to presenting or not a chronic disease.

^b^ Adjusted PR by age and region, comparing use of health services according to presenting or not a chronic disease.

^c^ Adjusted PR by age and region, comparing use of health services according to sex.

Table 2 presents strong gradients of increased use of health services with the increase in the number of diseases, and the associations are found even in the presence of only one NCD. The associations showed great magnitude, especially considering the indicator failing to perform activities for health reasons (PR = 6.62, comparing four or more diseases with no NCD). The use of health services and hospitalization in the last 12 months were respectively 3.4 and 3.3 times greater in the presence of four or more NCDs. The associations tend to be similar among men and women. However, the association among use of services and hospitalization is higher in males, in the presence of three chronic diseases.

**Table 2:** Use of health services and failing to perform activities for health reasons according to the number of chronic noncommunicable diseases (NCD). National Health Survey, 2013.

Variable	Population without NCD	Population with NCD – number of morbidities				Prevalence ratios^a^			
		
	(0)	1	2	3	4 or more	1/0	2/0	3/0	4/0
Total									
Use of health services in the last two weeks (%) J14	10.87	20.06	26.65	37.28	46.56	1.72 (1.58–1.87)	2.17 (1.97–2.39)	2.91 (2.62–3.23)	3.46 (3.09–3.87)
Hospitalization in the last 12 months (%) J37	4.91	7.15	10.44	13.57	19.30	1.38 (1.20–1.58)	1.94 (1.68–2.24)	2.44 (2.03–2.93)	3.33 (2.75–4.04)
Failing to perform activities in the last two weeks for health reasons (%) J2	4.06	9.06	13.31	21.16	28.23	2.18 (1.92–2.48)	3.19 (2.79–3.65)	5.03 (4.25–5.95)	6.62 (5.56–7.90)
Medical consultation in the last 12 months (%) J11 = 01	65.36	79.95	89.15	92.78	96.45	1.19 (1.16–1.21)	1.29 (1.26–1.32)	1.32 (1.28–1.35)	1.33 (1.30–1.37)
Males									
Use of health services in the last two weeks (%) J14	8.31	16.24	21.69	38.10	43.02	1.79 (1.55–2.07)	2.21 (1.87–2.62)	3.71 (3.09–4.47)	4.00 (3.15–5.07)
Hospitalization in the last 12 months (%) J37	3.05	6.16	9.55	15.55	23.50	1.71 (1.35–2.15)	2.39 (1.89–3.03)	3.60 (2.65–4.90)	5.18 (3.58–7.49)
Failing to perform activities in the last two weeks for health reasons (%) J2	3.63	8.31	11.12	17.89	26.00	2.25 (1.83–2.77)	3.05 (2.14–3.84)	4.92 (3.64–6.66)	7.19 (5.09–10.15)
Medical consultation in the last 12 months (%) J11 = 01	56.96	73.39	84.47	91.15	92.13	1.25 (1.21–1.29)	1.39 (1.33–1.45)	1.47 (1.41–1.54)	1.46 (1.37–1.55)
Females									
Use of health services in the last two weeks (%) J14	13.67	23.17	29.97	36.85	47.78	1.66 (1.50–1.84)	2.11 (1.89–2.36)	2.57 (2.26–2.91)	3.29 (2.90–3.73)
Hospitalization in the last 12 months (%) J37	6.94	7.95	11.03	12.54	17.87	1.20 (1.01–1.43)	1.73 (1.45–2.06)	2.00 (1.58–2.53)	2.89 (2.32–3.62)
Failing to perform activities in the last two weeks for health reasons (%) J2	4.53	9.67	14.77	22.86	28.99	2.13 (1.80–2.52)	3.26 (2.75–3.86)	5.06 (4.14–6.20)	6.46 (5.26–7.93)
Medical consultation in the last 12 months (%) J11 = 01	74.51	85.27	92.27	93.63	97.93	1.14 (1.11–1.17)	1.23 (1.20–1.25)	1.24 (1.20–1.28)	1.29 (1.26–1.32)

^a^ PR adjusted by age, sex, and region.

Regarding health insurance, the prevalence of use of health services was higher in individuals with NCDs, considering all the indicators used in the study (Table 3). Individuals without health insurance, with or without NCD, had lower prevalence of use of services, hospitalization, and medical consultation. To the “failing to perform activities for health reasons” indicator, the prevalence was 22% greater in the population without health insurance only for individuals with NCDs (Table 3).

**Table 3:** Use of health services and failing to perform activities for health reasons according to health insurance. National Health Survey, 2013.

Variable	Population with NCD		Population without NCD		Crude PR^a^	Adjusted PR^b^	95%CI
	
	%	PR (95%CI)^c^	%	PR (95%CI)^c^			
Possess health insurance							
Use of health services in the last two weeks	29.91	1	13.95	1	2.14	1.93	1.70–2.18
Hospitalization in the last 12 months	10.87	1	6.03	1	1.80	1.54	1.22–1.93
Failing to perform activities in the last two weeks for health reasons	10.98	1	4.21	1	2.61	2.47	1.98–3.07
Medical consultation in the last 12 months	93.40	1	82.20	1	1.14	1.11	1.09–1.14
Do not possess health insurance							
Use of health services in the last two weeks	24.10	0.86 (0.79–0.93)	9.96	0.76 (0.67–0.86)	2.42	2.06	1.88–2.25
Hospitalization in the last 12 months	9.15	0.85 (0.74–0.98)	4.58	0.76 (0.63–0.91)	2.00	1.76	1.56–1.99
Failing to perform activities in the last two weeks for health reasons	13.62	1.22 (1.07–1.40)	4.02	0.91 (0.73–1.12)	3.39	2.93	2.58–3.32
Medical consultation in the last 12 months	81.93	0.90 (0.89–0.91)	60.36	0.76 (0.74–0.78)	1.36	1.28	1.25–1.31

^a^ Crude PR, comparing use of health services according to presenting or not a chronic disease.

^b^ Adjusted PR by age and region, comparing use of health services according to presenting or not a chronic disease.

^c^ Adjusted PR by age and region, comparing use of health services according to health insurance.

The population with NCD uses more health services than those without NCD at all educational levels studied (Table 4). It is also possible to observe that by comparing the use of services in the extreme education levels (illiterate/some elementary school with higher education degree), those who have studied less and reported a NCD had higher prevalence of failing to perform activities for health reasons (PR = 1.41; 95%CI 1.16–1.72) and lower prevalence of medical consultation in the last 12 months (PR = 0.91; 95%CI 0.90–0.94). People without NCD presented lower prevalence of use of health services and medical consultation in the lower education levels (PR = 0.81; 95%CI 0.69–0.97 and PR = 0.75; 95%CI 0.72–0.78, respectively).

**Table 4:** Use of health services and failing to perform activities for health reasons according to education level. National Health Survey, 2013.

Variable	Population with NCD		Population without NCD		Crude PR^a^	Adjusted PR^b^
	
	%	PR (95%CI)^c^	%	PR (95%CI)^c^		
Illiterate/Some elementary school						
Use of health services in the last two weeks	26.28	0.91 (0.81–1.03)	10.50	0.81 (0.69–0.97)	2.50	2.28 (2.03–2.57)
Hospitalization in the last 12 months	10.63	1.04 (0.85–1.28)	5.08	1.22 (0.94–1.59)	2.09	2.05 (1.74–2.43)
Failing to perform activities in the last two weeks for health reasons	15.09	1.41 (1.16–1.72)	4.96	1.19 (0.89–1.59)	3.04	2.95 (2.46–3.55)
Medical consultation in the last 12 months (%) J11 = 01	84.42	0.91 (0.90–0.94)	56.55	0.75 (0.72–0.78)	1.49	1.43 (1.38–1.48)
Elementary school/Some high school						
Use of health services in the last two weeks (%) J14	23.13	0.86 (0.74–1.00)	9.91	0.82 (0.68–0.99)	2.33	2.18 (1.86–1.48)
Hospitalization in the last 12 months (%) J37	8.73	0.96 (0.74–1.23)	4.81	1.10 (0.83–1.46)	1.82	1.63 (1.26–2.11)
Failing to perform activities in the last two weeks for health reasons (%) J2	11.86	1.20 (0.95–1.52)	3.62	0.85 (0.63–1.15)	3.28	3.22 (2.48–4.19)
Medical consultation in the last 12 months (%) J11 = 01	83.23	0.93 (0.90–0.96)	63.08	0.85 (0.81–0.88)	1.32	1.26 (1.21–1.32)
High School/Some higher education						
Use of health services in the last two weeks (%) J14	24.75	0.94 (0.82–1.07)	10.62	0.85 (0.73–0.98)	2.33	2.21 (1.96–2.49)
Hospitalization in the last 12 months (%) J37	8.36	0.95 (0.75–1.21)	4.90	1.07 (0.83–1.39)	1.71	1.58 (1.30–1.92)
Failing to perform activities in the last two weeks for health reasons (%) J2	10.88	1.12 (0.91–1.38)	3.49	0.82 (0.65–1.04)	3.12	3.00 (2.49–3.61)
Medical consultation in the last 12 months (%) J11 = 01	84.08	0.95 (0.92–0.97)	68.73	0.90 (0.87–0.93)	1.22	1.20 (1.16–1.23)
Higher education degree						
Use of health services in the last two weeks (%)	27.84	1	13.74	1	2.03	1.99 (1.71–2.32)
Hospitalization in the last 12 months (%)	9.15	1	4.70	1	1.95	1.91 (1.44–2.54)
Failing to perform activities in the last two weeks for health reasons (%)	9.83	1	4.17	1	2.36	2.36 (1.80–3.09)
Medical consultation in the last 12 months (%)	91.04	1	79.21	1	1.15	1.14 (1.11–1.18)

^a^ Crude PR, comparing use of health services according to presenting or not a chronic disease.

^b^ Adjusted PR by age and region, comparing use of health services according to presenting or not a chronic disease.

^c^ Adjusted PR by age and region, comparing use of health services according to education level.

## DISCUSSION

The results show that, of the 45% of adults who reported having a NCD, 25% used health services in the last two weeks. People with NCD use the services twice as much than adults without NCD. NCD carriers reported more hospitalization, failed to perform activities in the last two weeks for health reasons and attended more medical consultations in the last 12 months. The population with NCD, for both sexes, uses more health services than the population without NCD. The study also identified that women use the services more, both for consultations and hospitalizations, and reported more frequently of failing to perform activities for health reasons in the last two weeks. The increase in comorbidities also increased the demand for health services. The use of health services according to education level showed that those who have studied less and reported having a NCD had higher prevalence of hospitalization in the last 12 months, higher prevalence of failing to perform activities for health reasons, and higher prevalence of medical consultation in the last 12 months. Individuals without health insurance, with or without NCD, had lower prevalence of use of services, hospitalization, and medical consultation, when compared to those with health insurance.

### Users with NCDs

NCD carries had used the health services more, i.e., the need felt by the user is the biggest motivator for the demand and use of health services. This may be explained by the demands of routine or intercurrence consultations, greater comorbidities, and also by other diseases or aggravations^2,10,i^. In fact, the use of the service is determined by the need perceived by users as a result of the health situation and their previous knowledge of their disease or health condition^11,12^. We highlight the causal relationship between disease and the use of health services, disease being the main responsible for the use of health services^6,7,12^.

A study that analyzed data from the Brazil National Household Sample Survey in 2003 and 2008 indicate that individuals with chronic diseases seek health services more^13^. PNS confirmed that more motivation for using the service is linked to the presence of diseases. Therefore, it is important to monitor the use of the services in this population, to establish strategies to adapt the demand and consumption of these services^13^.

Travassos et al.^5^ consider the fact the user feel susceptible to a given health problem is an important motivator for using the services. Some authors emphasize that the severity of the disease and the belief in the benefits from the treatment or preventive action are important elements for the use of health services^5,14^. According to Travassos et al.^5^, this situation is due to the subjective perception of the risk of having a disease and the perceived gravity, in addition to feelings and concerns regarding the consequences of the disease, such as death, pain, or disability, as well as worse living conditions.

### Number of NCDs

The PNS also identified increased use of services as the number of NCDs or comorbidities increased, which may be related to greater awareness of the severity of the disease, its risks, and health threats^5^.

### Sex

The sociodemographic characteristics of users are among the determinants of use of health services. The study identified that women use the services more, both in consultations and hospitalizations, and report more limitations due to NCDs. This increased consumption was already identified by other authors^5,13,15^, who attributed this usage to women’s higher perception of the symptoms and signs of disease and, consequently, increased demand for services, physicians, tests, and promotion and prevention practices. Women who are not NCD carriers also presented high use of health services due to the practices of promotion, other acute diseases, and pregnancy^5,13,15^.

### Health Insurance

Individuals with health insurance, with or without NCD, presented more use of services, hospitalization, and medical consultation. This result corroborates the data from the PNAD, which showed that people with private health insurance presented higher prevalence of medical consultation and hospitalization in the last 12 months, and of use of services in the last two weeks, as well as smaller proportions of restriction of activities, when compared to the social segment that did not have health insurance^13,16^.

Studies comparing users that only had access to the Brazilian Unified Health System (SUS) with beneficiaries of complementary health observed lower frequencies of medical consultations and screening examinations in the first group^17-19^. The use by complementary health users is of 5.1 consultations per beneficiary of insurance consultations a year^j^ – about double of the parameter per year provided for the general population (2 to 3 consultations per inhabitant/year)^k,l,m^. SUS has as its principles universal access, completeness and fairness, which ensures the use and access of services for the population with lower educational level, income, and no health insurance. However, differences in the use of services that benefit people who have health insurance remain. The population with health insurance may also have greater opportunity to access to services, by accessing the services of both SUS and complementary health^20^.

### Education Level

Studies conducted in developed countries found a higher prevalence of NCDs in populations with lower education level^13,17^. In Brazil, many chronic diseases present a social gradient that grows in the direction of the most socially vulnerable segments, as observed in this study. The two editions of the *Pesquisa Nacional por Amostragem de Domicílio* (PNAD – National Household Sampling Survey) (2003 and 2008)^13^ found lower use of health services and lower proportion of medical consultations in populations with lower education level. This pattern persisted in this study. Users with NCD and higher education used services more, compared to the less educated. We would like to highlight the iniquity on occurrence of NCDs in the population without insurance, which showed the highest degree of limitation in performing the activities in the two previous weeks, for reasons of health.

Among the limitations of the study is the cross-sectional design, which, although advantageous due to the speed and low cost, has disadvantages inherent to the study, such as the possibility of reverse causality. The use of self-reported diagnosis is also subjected to access to health services. In addition, this study used two different sections of the PNS: the randomly chosen resident answered about self-reported NCDs; and the use of health services (section J) was answered by one of the residents, not necessarily the chosen resident, which may bias the presented prevalence.

## CONCLUSIONS

NCD carriers make more use of health services, as well as women, people with higher number of comorbidities, with health insurance, and higher education level.

Investing in health systems is critical to improve the results of NCDs, which includes the strengthening of the health system, financing, governance, management, human resources in health, health information, and access to technologies and medicines^a,b,d^. Indicators of use of health services are important to assess health care quality, regarding access and use of the services by the different segments of the population. Knowing how NCD carriers use health services is essential to reduce access barriers and guide health policies, providing equity in access to resources, as well as guiding the design of policies directed at reducing vulnerabilities^d^.

## Footnotes

a World Health Organization. Health statistics and information systems: estimates for 2000-2012. Geneva: WHO; s.d. [cited 2014 Nov 3]. Available from: http://www.who.int/healthinfo/global_burden_disease/estimates/en/index1.html

b World Health Organization. Global status report on noncommunicable diseases 2010. Geneva: WHO; 2011 [cited 2014 Nov 3]. Available from: http://www.who.int/nmh/publications/ncd_report2010/en/

c World Health Organization. From burden to “best buys”: reducing the economic impact of non-communicable diseases in low- and middle-income countries: executive summary 2011. Geneva: World Health Organization; World Economic Forum; 2011 [cited 2014 Nov 3]. Available from: http://www.who.int/nmh/publications/best_buys_summary/en/

d World Health Organization. Global Action Plan for the Prevention and Control of NCDs 2013-2020. Geneva: WHO; 2013 [cited 2014 Oct 20]. Available from: http://www.who.int/nmh/events/ncd_action_plan/en/

e Organização das Nações Unidas no Brasil – ONU-BR. 17 objetivos para transformar nosso mundo: objetivo 3: assegurar uma vida saudável e promover o bem-estar para todos, em todas as idades. Brasília (DF); 2015 [cited 2016 Jan 5]. Available from: https://nacoesunidas.org/pos2015/ods3/

f World Health Organization. Political Declaration of the High-level Meeting of the General Assembly on the Prevention and Control of Non-communicable Diseases. In: Sixty-sixth Session of the United Nations General Assembly, Sept 2011, New York. Resolution 66/2. New York: UN; 2011 [cited 2014 Nov 3]. Available from: http://www.who.int/nmh/events/un_ncd_summit2011/political_declaration_en.pdf?ua=1

g Instituto Brasileiro de Geografia e Estatística. Pesquisa Nacional de Saúde: 2013. Acesso e utilização dos serviços de saúde. Acidentes e Violências. Rio de Janeiro: Instituto Brasileiro de Geografia e Estatística; 2015. 106 p. http://www.ibge.gov.br/home/estatistica/populacao/pns/2013_vol3/default_microdados.shtm

h The microdata (free access) are available from: http://www.ibge.gov.br/home/estatistica/populacao/pns/2013_vol3/default_microdados.shtm

i Cesar CLG, Goldbaum M. Uso de serviços de saúde. In: Cesar CLG, Carandina L, Alves MCGP, Barros MBA, Goldbaum M. Saúde e condição de vida em São Paulo: inquérito multicêntrico de saúde no Estado de São Paulo – ISA-SP. São Paulo: Faculdade de Saúde Pública da USP; 2005. p.185-98.

j Instituto de Estudos de Saúde Suplementar. Guia de Saúde Suplementar. São Paulo: IESS; 2013 [cited 2017 Jan 24]. Available from: http://www.iess.org.br/guia_iess_tela.pdf

k Ministério da Saúde (BR). Portaria nº 3.295, de 13 de novembro de 1998. Aprova o Manual para Organização da Atenção Básica no Sistema Único de Saúde, e dá outras providências. Brasília (DF); 1998 [cited 2017 Jan 25]. Available from: http://bvsms.saude.gov.br/bvs/saudelegis/gm/1998/prt3925_13_11_1998_rep.html

l Ministério da Saúde (BR). Programa de Atenção Básica. Portaria nº 832 de 28 de junho de 1999. Regulamenta o processo de acompanhamento e de avaliação da Atenção Básica à Saúde, quanto aos resultados a serem alcançados e quanto à oferta de serviços financiados pelo Piso de Atenção Básica – PAB, conforme expresso na Norma Operacional Básica do SUS 01/96 e no Manual para Organização da Atenção Básica à Saúde e dá outras providências. *Diario Oficial União*. 29 Jun 1999. DO 122-E.

m Ministério da Saúde (BR), Programa de Atenção Básica. Portaria nº12 de 07 de janeiro de 2000. Dá continuidade ao processo de avaliação da Atenção Básica, estabelecido pela Portaria GM/MS n.º 3.925, de 13 de novembro de 1998 e dá outras providências. *Diario Oficial União*. 12 Jan 2000. DO 8-E.
